# Minimally invasive management for multifocal pelvic retroperitoneal malignant paraganglioma: a neuropelveological approach

**DOI:** 10.1186/s12905-022-01969-7

**Published:** 2022-09-18

**Authors:** Giulia Zaccaria, Giuseppe Cucinella, Mariano Catello Di Donna, Giuseppe Lo Re, Giuseppe Paci, Antonio Simone Laganà, Vito Chiantera

**Affiliations:** 1 Unit of Gynecologic Oncology, ARNAS “Civico – Di Cristina – Benfratelli”, Palermo, Italy; 2 Department of Surgical, Oncological and Oral Sciences (Di.Chir.On.S.), University of Palermo, Palermo, Italy; 3 Department of Biomedicine, Neuroscience and Advanced Diagnostics, University of Palermo, Palermo, Italy; 4 Department of Health Promotion, Mother and Child Care, Internal Medicine and Medical Specialties (PROMISE), University of Palermo, Palermo, Italy

**Keywords:** Paraganglioma, Neuropelveology, Robotic surgery, Minimally invasive gynecological surgery, Surgery, Oncology, Cancer, Gynecology

## Abstract

**Background:**

Pheochromocytoma and Paraganglioma (PGL) are rare neuroendocrine tumors, with an estimated incidence of about 0.6 cases per 100.000 person/year. Overall, 3–8% of them are malignant. These tumors are characterized by a classic triad of symptoms (headaches, palpitations, profuse sweating) due to hypersecretion of catecholamines. Despite several advantages of minimally invasive surgery (MIS) for PGL debulking, the surgical approach is not standardized yet. In this scenario, we aimed to report a case of a multiple recurrent PGL with metastatic retroperitoneal localization involving the pelvic sidewall, excised with MIS.

**Case presentation:**

We performed complete laparoscopic-assisted neuronavigation (LANN technique) with isolation of the sacral routes and the sciatic nerve to obtain complete exposure of the main anatomic landmarks. Robotic surgery was used to perform neurolysis of sacral plexus, and partial resection of left splanchnic nerves was needed. After the resection of the first mass, extensive neurolysis of all sacral routes, obturator nerve, pudendal nerve till the entrance of the pudendal (Alcock) canal, and sciatic nerve was performed. Finally, the mass was identified after trans gluteal incision and dissection of the maximum gluteal muscle, and a partial resection of the superior gluteal nerve and slicing of the sciatic nerve were needed to obtain a radical excision of the mass. Then neurorrhaphy of the sectioned nerve fibers of the superior gluteal nerve was performed, and nerve protection was obtained using a collagen nerve wrap. After 18 months of follow-up, the patient is free of disease at the MRI imaging and 123I-metaiodobenzylguanidine scintigraphy.

**Conclusions:**

Minimally invasive gynecological surgery with neuropelveological approach could be considered as a feasible option in case of multifocal pelvic retroperitoneal malignant paraganglioma of the pelvic side wall.

## Background

Pheochromocytoma (PH) and Paraganglioma (PGL) are rare neuroendocrine tumors, with an estimated incidence of about 0.6 cases per 100.000 person/year. Overall, 3–8% of those cases are malignant [1]. PH and PGL are characterized by a classic triad of symptoms (headaches, palpitations, profuse sweating) due to hypersecretion of catecholamines [2]. Anatomical location is used to differentiate the two tumor types: PH is an adrenal tumor, while PGL is an extra-adrenal tumor. The PGL affecting sympathetic system is most often located in the retroperitoneum, along with the thoracolumbar paravertebral region and the bladder; conversely, the involvement of the pelvic sidewall is very rare [3]. Complete surgical excision of the PGL is the first-line approach, while metastatic or recurrent tumors often require further adjuvant treatments. Despite several advantages of Minimally Invasive Surgery (MIS) for PGL debulking, the surgical approach is not standardized yet. Only a few studies reporting the resection of pelvic isolated retroperitoneal PGL through MIS have been published and, to the best of our knowledge, multiple recurrent PGL with metastatic localization resected with an endoscopic approach has not been described so far.

In this scenario, we aimed to report a case of a multiple recurrent PGL with metastatic retroperitoneal localization involving the pelvic sidewall, excised with MIS.

## Case presentation

A nulliparous 29-year-old woman was referred to our Institution due to a second paraganglioma recurrence with systemic symptoms. She did not have family history of PH/PGL syndrome. The first diagnosis of PGL was in 2003, after an urgent laparotomic resection of a pelvic mass with concomitant heart failure. In 2006, pelvic Magnetic Resonance Imaging (MRI) showed the first recurrence with multiple pelvic nodules. Laparotomic debulking was performed, though a non-complete resection of the disease was reported. Post-operative MRI confirmed the persistence of disease in the left pelvic wall, left parametrial tissue and paravesical space. The patient received six cycles of adjuvant chemotherapy with Etoposide, Doxorubicin and Cisplatin. In November 2020, the patient experienced an exacerbation of hypertension and lipothymia. The patient also complained of hypertensive peaks in supine position.

The pelvic bimanual evaluation allowed to identify a left gluteal mass and a solid mass located in the left pararectal space; invasion of the vaginal and rectal mucosa was not apparent. Interestingly, the compression of the left gluteal mass led to hypertensive peak. In addition, investigation of the pelvic nerves was performed based on neuropelveological criteria [4]. The analysis of the trigger points, performed through the transrectal and transvaginal palpation of the sacral plexus, was negative. Laboratory tests revealed elevated epinephrine levels on blood and urine samples. CA125, CA19-9, CA15-3 and CEA biomarkers were within normal range.

**Fig. 1:**
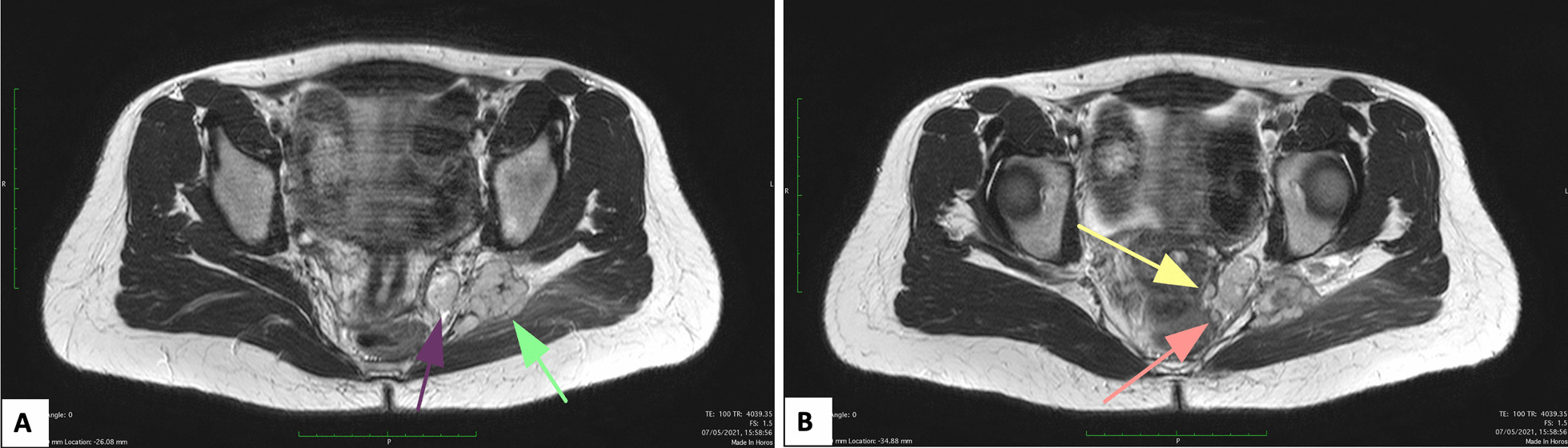
Magnetic Resonance Imaging (MRI) axial T1 weighted sequencies of the pelvis showing multiple nodules: **A** irregular nodule in gluteal region between the large gluteal muscle and the piriformis muscle (green arrow). Double nodule at the apex of ischiorectal fossa which is contiguous to the largest gluteal nodule (purple arrow); **B** two little nodules in the left obturator space, next to distal anal canal (yellow and pink arrow)

**Fig. 2:**
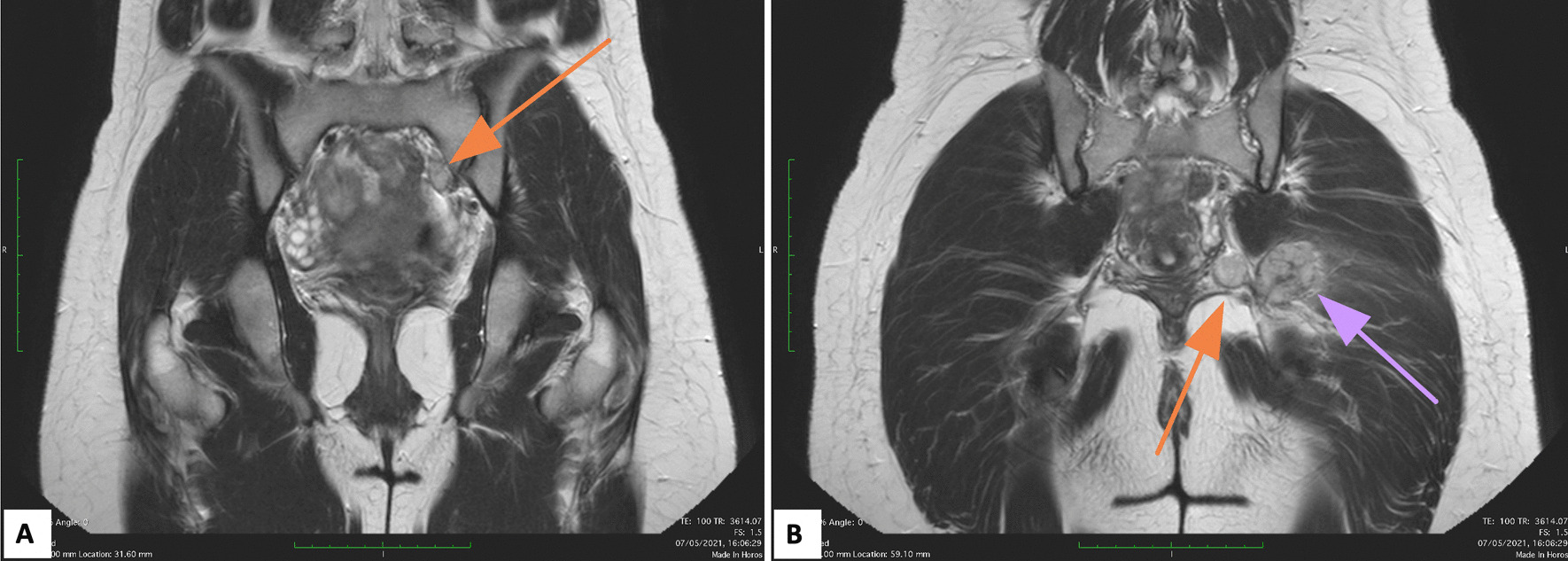
Magnetic Resonance Imaging (MRI) coronal T1 weighted sequencies of the pelvis: **A** nodule (15 mm) at the left external iliac level (orange arrow); **B** gluteal nodule (purple arrow) and two contiguous nodules in the ischiorectal fossa (orange arrow)

Pre-operative MRI showed multiple pelvic nodules (Fig. 1): a 4.5 × 2.7 × 3 cm mass in the left gluteal region, with suspicious involvement of the sciatic nerve; two contiguous nodules, 2 × 1.7 × 2 cm and 3 × 1.7 × 2 cm, respectively, in the ischiorectal fossa; an 8 mm nodule in the obturator space; a 15 cm mass in the left external iliac region and a 1.4 × 1.7 × 1.5 cm nodule in the vesical-vaginal septum (Fig. 2).

After discussion of the case within our multidisciplinary tumor board, including the cardiological and anesthesiologic teams, the patient was scheduled for robotic resection of the pelvic nodules and transgluteal resection of the gluteal mass.

**Fig. 3:**
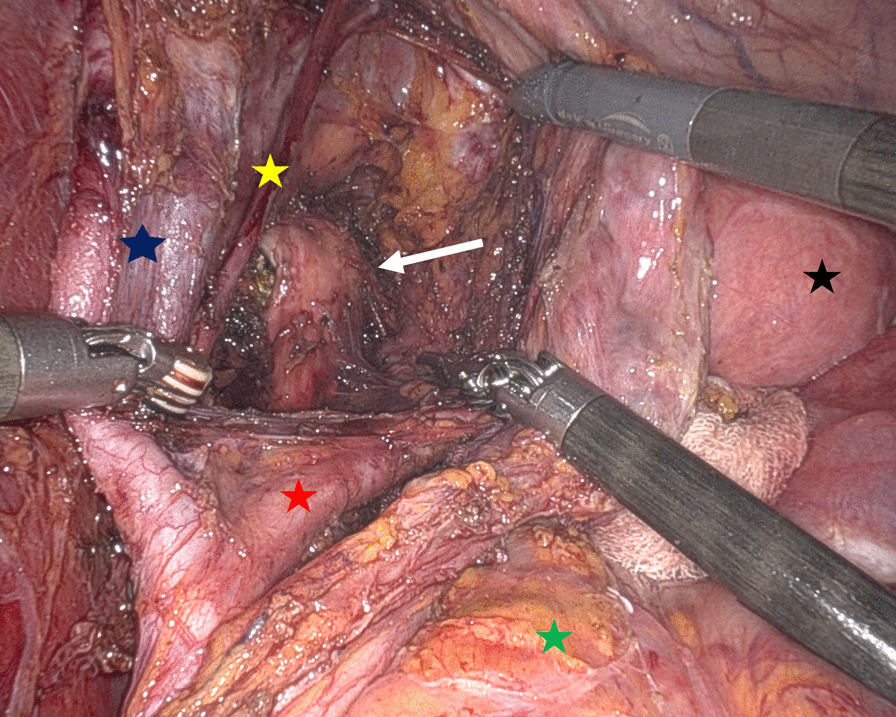
Left retroperitoneal view of sacral plexus (yellow star = obturator nerve; black star = uterus; green star = sigma-rectum; blue star = external iliac vessels; red star = internal iliac vessels; white arrow = sciatic nerve)

The patient underwent preoperative preparation with alpha (phenoxybenzamine) and beta-blocker (metoprolol) agents the last two weeks before the surgery, in order to stabilize the pressure and avoid abrupt arterial pressure fluctuations during the surgery. A robot-assisted approach was performed using a 4-armed Da Vinci Si platform (Intuitive Surgical Inc., Sunnyvale, CA, USA). The surgical exploration revealed normal upper abdominal organs, uterus and both ovaries. After access of the retroperitoneum and isolation of the left ureter, the inspection of the pelvis showed a solid bilobed mass in the context of the lateral and left posterior parametrium, adherent to the internal iliac vessels and the parietal endopelvic fascia. Then the dissection of the tumour started, without direct manipulation, to avoid hypertensive crisis. A complete laparoscopic-assisted neuronavigation (LANN technique) was performed, following standardized approach [5, 6], with isolation of the sciatic nerve and sacral routes, aimed to obtain the complete exposure of the main anatomic landmarks (Fig. 3).

**Fig. 4:**
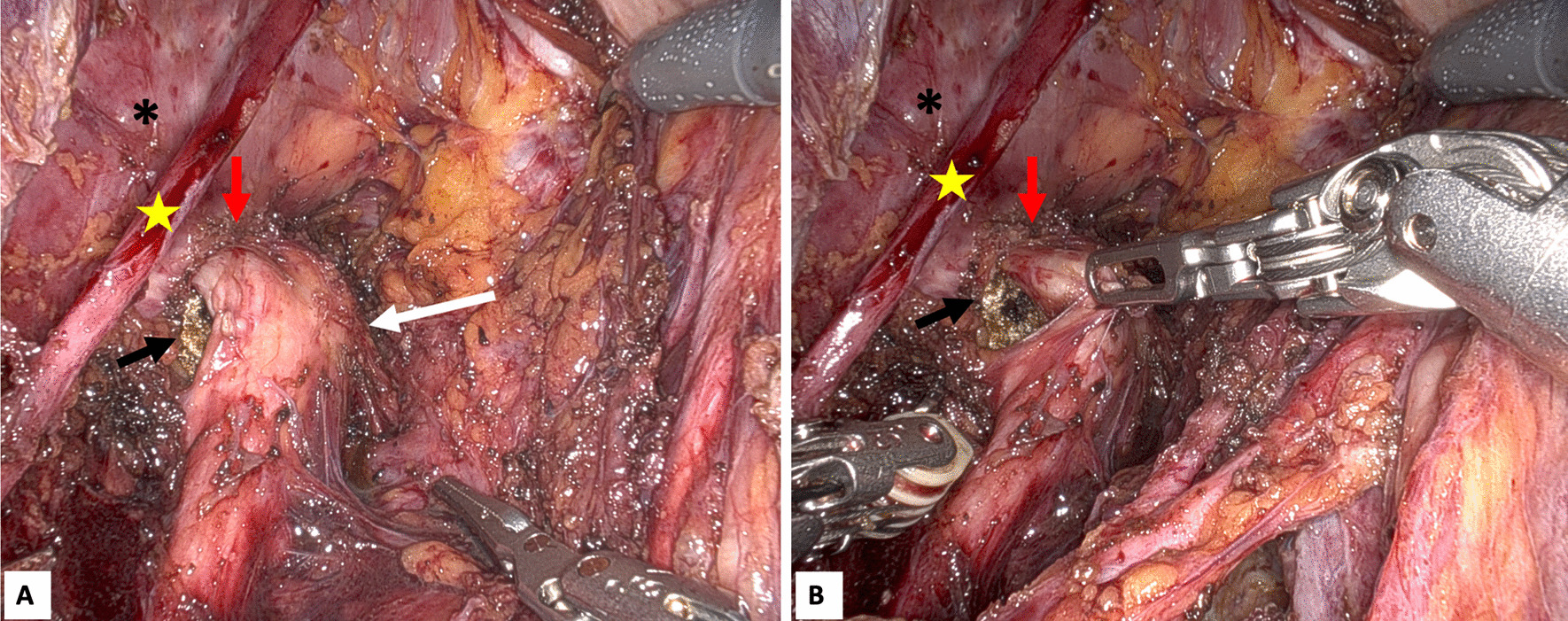
Neurolysis of the left sciatic nerve: **A** Complete exposure of the sciatic nerve; **B** Marked sciatic nerve before proceeding with trans gluteal approach (yellow star = obturator nerve; black star = pelvic side wall; red arrow = Alcock canal; white arrow = sciatic nerve; black arrow = marked sciatic nerve)

Once the inferior hypogastric plexus and the sacral plexus were exposed, a partial involvement of the autonomic pelvic innervation was found. Aiming to obtain a radical excision of the nodule, neurolysis of sacral plexus and partial resection of left splanchnic nerves were needed. After the resection of the first mass, extensive neurolysis of all sacral routes, obturator nerve, pudendal nerve till the entrance of the pudendal (Alcock) canal, and sciatic nerve was performed (Fig. 4a), as previously described [7].

In the left obturator space, two centimetric nodules strongly adherent to the obturator nerve and vein were removed with concomitant dissection of enlarged obturator lymph nodes. The obturator nerve and vein were safely spared during the debulking procedures.

Subsequently, the vesical-vaginal space was exposed after completely dissecting off the vesical peritoneum, and infiltration of the vaginal and bladder wall was detected. To obtain a complete excision of the nodule, partial centimetric resections of the vaginal and bladder wall were needed, with subsequent single-stiches suture. Filling the bladder with 200 cc of water showed no leakage or diverticula. All specimens were removed within endobag through the 12-mm assistant port.

Once the resection of all pelvic nodules was completed, the patient was placed in prone position. Intraoperative trans-gluteal ultrasound confirmed the 6 cm gluteal nodule in the context of the muscle fibers. The mass was identified after trans gluteal incision and dissection of the maximum gluteal muscle. The tumor was fixed with partial infiltration of the superior gluteal nerve and sciatic nerve, previously marked during the robotic neurolysis (Fig. 4b). A partial dissection of the superior gluteal nerve and slicing of the sciatic nerve were needed to obtain a radical excision of the mass. Then neurorrhaphy of the sectioned nerve fibers of the superior gluteal nerve was performed, and nerve protection was obtained using a collagen nerve wrap. During the operation, the patient presented only slight blood pressure fluctuation following manipulation of the nodules, easily controlled with intravenous α-adrenergic blocker (phentolamine) and β-adrenergic blockers. The procedure was completed by robotic surgery, without complications. Operating time was 480 min, and estimated blood loss was 100 mL. Patient was discharged after 7 days, with an uneventful post-operative period. Final histopathological report confirmed a malignant paraganglioma with poorly differentiated cells [8] with partial vascular invasion.

Immunohistochemistry showed strong positivity for chromogranin, synaptophysin and neuron-specific enolase. Conversely, tumor cells were immunohistochemically negative for pan-cytokeratin and S100. Proliferation index was 20%, measured as Ki67 expression. Surgical margins were negative. Two of the nine lymph nodes removed were positive for the presence of metastatic cells.

A clinical follow-up 2 weeks postoperatively showed normal blood pressure and absence of hypertensive crisis. The urinary catheter was removed 30 days after the surgery. After one month, the patient denied any gait disturbance or tenderness to palpation in the area of the left buttock. The patient presented a normal bilateral hip abduction.

A new 24-h urine collection showed normal amounts of catecholamines. Two months after the surgery, a metaiodobenzylguanidine nuclear scan test was performed, showing no abnormal uptake. After discussion in our multidisciplinary tumor board, the patient underwent clinical observation with imaging and laboratory tests. After 18 months of follow-up, the patient was free of disease at the MRI imaging and 123I-metaiodobenzylguanidine scintigraphy. No new symptoms or signs were reported.

The patient signed informed consent to allow data collection for research purpose and the publication of the case. This article conforms the Consensus-based Clinical Case Reporting (CARE) Guideline [9], validated by the Enhancing the QUAlity and Transparency Of health Research (EQUATOR) Network. Considering the anonymized reporting of the case, a formal Institutional Review Board approval was waived.

## Discussion and conclusions

To the best of our knowledge, the first reported case of a multiple recurrence malignant PGL with retroperitoneal pelvic multifocal localization involving the pelvic side wall. Moreover, the resection was carried out totally through MIS, combining robotic-laparoscopic assisted procedures with a transgluteal approach. Although the complexity of the surgery, a complete resection of all the nodules was achieved without intraoperative complications.

Despite most of the extra-adrenal PGL have low malignant potential, in rare cases a malignant pattern has been observed [10]. Considering its rarity, there are no definitive criteria on histology to clearly differentiate benign from malignant PGL, and malignancy is determined by recurrence or distant metastasis [11]. To date, the only case of malignant recurrence available in the literature is a PGL of the bladder with metastatic pelvic lymph nodes [12]: nevertheless, the surgical approach for the tumor resection has not been reported. Moreover, the pelvic side wall involvement is a very rare scenario for neuroendocrine tumors. In literature, only one report described a case of paraganglioma of the left pelvic wall, abutting the left lateral wall of the urinary bladder, and removed by laparotomic approach [13]. In this scenario, our report describes the first MIS management for PGL of the pelvic side wall, allowing us to highlight the feasibility of this approach as we already reported for other disease of the same anatomical area [14].

The uniqueness of our report is also represented by the multifocal localization, with the presence of a metastasis in the context of the maximum gluteal muscle infiltrating the superior gluteal nerve. Despite the pre-operative imaging represented an important step for evaluation of the involved retroperitoneal structures and extension of the disease [15], the neuropelvic anatomic assessment helped us to obtain a better identification of the involvement of the pelvic nerves by the nodules. In the current case, the absence of somatic pain and motor dysfunction helped us to exclude a massive infiltration of the sacral routes. Moreover, the investigation of the pelvic nerves by vaginal and rectal examination guided us to identify a mass with involvement of the pelvic side wall and concomitant release of catecholamine and hypertension peak during the compression. Therefore, the wide knowledge of the pelvic neuroanatomy was essential to define the location of the nodule and rule out a possible extensive involvement of the sacral plexus. Indeed, the neuropelveology could be considered a new potential tool for the preoperative mapping of the lesions. From this perspective, the results of neuropelveological examination allows for a better and tailored treatment, improving diagnostic accuracy and lesion localization.

Based on clinical and radiological data, the neurogenic lesions were safely accessible thought a combined approach (robotic and transgluteal), although extensive neurolysis of sacral plexus and partial resection of left pelvic parasympathetic innervation and superior gluteal nerve were needed. Overall, several factors contributed to make the surgical management very challenging: among the most important, the multifocal and unusual location of the nodules, the partial infiltration of major vascular structures and pelvic nerves, and the adhesions caused by previous laparotomic surgery with incomplete excision of the disease. For these reasons, in our opinion the support of the robotic system in this complex procedure played a pivotal role for the precise dissection of the highly vascularized tumor with a magnified field of vision and 3D imaging, and allowed minimization of postoperative morbidity and hospital stay. Although our case showed the feasibility of the first robotic resection of a PGL recurrence, other groups reported MIS for PGL and PH as a safe approach: Walz et al. [10] described their successful experience in the largest case series of neuroendocrine tumors excised by using all varieties of endoscopic approaches (transabdominal laparoscopy, retroperitoneoscopic and extraperitoneal approaches). No standardized post-operative treatment guidelines were available, since this condition could be considered extremely rare. Chemotherapy, target therapy, or radiation therapy could be considered as adjuvant treatments after surgical debulking. However, these treatments do not seem to improve survival in patients with neuroendocrine tumors [16].

The decision of clinical follow-up after surgery was discussed in our multidisciplinary tumor board, based on the complete resection achieved and the negative post-operative imaging. Despite the low incidence of this rare entity, each retroperitoneal pelvic mass with concomitant symptoms of catecholamine excess should be potentially considered in the differential diagnosis as a malignant PGL [17]. Nevertheless, even in case of pelvic side wall involvement with nerve infiltration, a surgical excision with a robot-assisted laparoscopic approach seems feasible and safe. Despite the rare incidence of malignant retroperitoneal PGL, further studies are needed to evaluate the optimal method to diagnose and manage this challenging disease.

## Data Availability

All data generated or analysed during this study are included in this published article.

## Abbreviations

PH: Pheochromocytoma
PGL: Paraganglioma
MIS: Minimally invasive surgery
MRI: Magnetic resonance imaging
